# Inhibition of miR-4640-5p alleviates pulmonary hypertension in chronic obstructive pulmonary disease patients by regulating nitric oxide synthase 1

**DOI:** 10.1186/s12931-023-02387-5

**Published:** 2023-03-24

**Authors:** Zhao Yang, Ping Li, Qun Yuan, Xi Wang, Hong-Hong Ma, Bing Zhuan

**Affiliations:** 1 Department of Respiratory Medicine, Suzhou Science & Technology Town Hospital, Suzhou, 215153 Jiangsu China; 2 Department of Respiratory Medicine, People’s Hospital of Ningxia Hui Autonomous Region, The Affiliated Hospital of NingXia Medical University, Ningxia, Yinchuan, 750001 China

**Keywords:** Pulmonary hypertension, COPD, PASMC, miR-4640-5p, NOS1

## Abstract

**Background:**

Pulmonary hypertension (PH) is a devastating disease characterized by vasoconstriction and vascular remodeling, leading to right ventricular failure and death. PH is a common complication of chronic obstructive pulmonary disease (COPD). Accumulating evidence demonstrate that microRNAs participate in the pathobiology of PH in COPD patients. In this study, we aimed to evaluate the expression and function of microRNA-4640-5p (miR-4640-5p) in PH.

**Methods:**

The mRNA and protein levels were determined by quantitative polymerase chain reaction (qPCR) and western blot, separately. Functional assays and western blot were performed to determine the effects of miR-4640-5p and NOS1 on cell growth, migration. Besides, the dual-luciferase reporter assays were used to validate miR-4640-5p and NOS1 interactions.

**Results:**

We found that miR-4640-5p expression was significantly higher in the lung tissues of COPD-PH patients than in the healthy controls while higher expression of miR-4640-5p was correlated with more severe COPD-PH. By using pulmonary artery smooth muscle cell (PASMC) in in vitro assays, we demonstrated that inhibition of miR-4640-5p suppressed cell proliferation and migration of PASMC via regulating mTOR/S6 signaling. Bioinformatics analysis and validation experiments revealed that nitric oxide synthase 1 (NOS1) was a direct downstream target of miR-4640-5p. Overexpression of NOS1 partially antagonized the effect of miR-4640-5p in regulating PASMC cell proliferation and migration. In addition, our findings suggested that miR-4640-5p/NOS1 axis regulated mitochondrial dynamics in PASMCs. Furthermore, in the hypoxia-induced PH rat model, inhibition of miR-4640-5p ameliorated PH with reduced right ventricular systolic pressure and Fulton index.

**Conclusions:**

miR-4640-5p regulates PH via targeting NOS1, which provides a potential diagnostic biomarker and therapeutic target for COPD-PH patients.

**Supplementary Information:**

The online version contains supplementary material available at 10.1186/s12931-023-02387-5.

## Background

Pulmonary hypertension (PH) is a devastating disease featured with elevated pulmonary vascular resistance, vasoconstriction and vascular remodeling, leading to right ventricular failure and death [1, 2]. PH is a common complication of chronic obstructive pulmonary disease (COPD) [3]. The therapeutic treatment for PH associated with COPD has been advanced significantly during the past decade [4, 5]. However, there is no cure treatment and current therapies only focus on vasodilation but not vascular remodeling [6]. Studies have demonstrated that pulmonary artery smooth muscle cells (PASMCs) play critical roles in thickening media and distal vessel muscularization [7]. Thus, it is of great importance to develop new therapeutic treatments targeting PASMC and prevent vascular remodeling in PH associated with COPD.

Accumulating evidence demonstrate that microRNAs play key functions in the pathobiology of PH in COPD patients, which could be utilized as biomarkers and therapeutic targets for the diagnosis and treatment of patients with COPD-PH [8, 9]. Liu et.al reported that high expression of microRNA-214 promoted the development of vascular remodeling in hypoxia-induced PH by targeting CCNL2 [10]. MiR-190a-5p was demonstrated to participate in the regulation of hypoxia-induced PH and could be utilized as a biomarker for diagnosis and prognosis in COPD-PH patients [11]. Similarly, increased expression of plasma miR-210 could serve as a diagnostic biomarker for COPD-PH [12]. MiR-4640-5p was found to interact with lncRNA OGFRP1 and regulate eIF5A expression in non-small cell lung cancer (NSCLC) [13]. However, the expression and function of miR-4640-5p in COPD-PH and its underlying mechanisms are not fully studied.

Nitric oxide is an endogenous pulmonary vasodilator that is synthesized by nitric oxide synthase (NOS), playing an important role in pulmonary vascular tone and vascular remodeling [14]. There are three different NOS isoforms which comprises neuronal NOS (nNOS, NOS1), inducible NOS (iNOS, NOS2) and endothelial NOS (eNOS, NOS3) [15]. NOS1 has been found to regulate pulmonary hypertension in animal models [16]. The mRNA and protein expression of NOS1 was significantly higher in lung tissues of smokers with COPD compared with nonsmoker controls [17]. How NOS1 expression was regulated in COPD-PH remains unclear.

In this study, we aimed to evaluate the expression of microRNA-4640-5p (miR-4640-5p) in the lung tissues of PH patients. The function of miR-4640-5p evaluated both in in vitro PASMC and in vivo hypoxia-induced rat PH model. Our findings suggest that miR-4640-5p regulates PH via targeting NOS1, which provides a potential diagnostic biomarker and therapeutic target for COPD-PH patients.

## Methods

### COPD-PH patient specimen

Lung tissues from COPD-PH patients or normal donors were from Suzhou Science & Technology Town Hospital. Patient and specimen information from experimental group and control group were listed in Additional file 1: Tables S1, Additional file 2: Table S2 and Additional file 3: Table S3. Informed consent was obtained from all participants. The study protocols were approved by the research ethics committee of Suzhou Science & Technology Town Hospital and were conducted in accordance with the principles expressed in the Declaration of Helsinki.

### Cell culture and treatment

Human primary pulmonary artery smooth muscle cells (PASMCs) were purchased from the ScienCell (California, USA) and cultured in the DMEM (Gibco) with the recommended vascular smooth muscle cell growth kit. Cells were cultured at 37 °C with 5% CO_2_, under normoxia (21% O_2_) or hypoxia (1% O_2_) condition.

### Transfection

MiR-4640-5p mimics (5′-UGGGCCAGGGAGCAGCUGGUGGG-3′), miR-4640-5p inhibitor (5′-CCCACCAGCUGCUCCCUGGCCCA-3′), and relative negative control (NC) mimics (#miR1N0000001-1-10; RIBOBIO) or NC inhibitor (#miR2N0000001-1-10; RIBOBIO) were purchased from RiboBio (Guangzhou, China). NOS1 overexpression vector were purchased from RiboBio (Guangzhou, China). Transfection was performed using AC0021M (AccuRef Scientific; Xi’an, Shaanxi, China) following the manufacturer’s instructions.

### CCK-8 assay

Cell counting Kit-8 (CCK-8) assay (#AC0011M, AccuRef Scientific) was performed to evaluate the PASMC proliferation following the manufacturer’s protocol. After culture for indicated time, CCK-8 reagent was added to the cell culture and the absorbance at 450 nm was measured to determine the cell viability.

### 5-Ethynyl-20-deoxyuridine (EdU) incorporation assay

PASMCs were cultured in 24-well plates for 24 h under hypoxia condition. DNA synthesis was analyzed by using an EdU assay kit reagent (RC0051M, AccuRef Scientific, China) for 2 h at 37 °C. Cells were also treated with DAPI solution for 5 min at room temperature.

### Cell migration assay

PASMCs were seeded into the upper chambers of the Transwell system (Corning 3422, USA) in serum-free medium. Medium with 10% FBS was added to the lower chamber. After 36 h incubation, cells that migrated through the membrane filter were fixed and stained with 0.5% crystal violet and then counted with a microscope.

### Wound-healing assay

PASMCs were seeded into six-well plates at a density of 1 × 10^6^ cells/well and cultured until reaching 80% confluence. An artificial scratch wound was generated using a sterile 200-μL tip. The streaked cells were washed with serum-free medium and cultured in complete medium for 36 h. Cell migration was recorded by an inverted microscope (20 × magnification).

### Hypoxia-induced PAH rat model

Animal experiments were performed according to the guidelines for the care and use of laboratory animals in Suzhou Science & Technology Town Hospital. The study protocol was reviewed and approved by the Institute Animal Care and Use Committees (IACUC) of Suzhou Science & Technology Town Hospital. Briefly, twenty male SPF Sprague–Dawley rats (250–275 g) were randomly divided into four groups (n = 5): Normoxia group, Hypoxia group, Hypoxia + anti-NC group, Hypoxia + anti-miR-4640-5p group. Rats in Hypoxia + anti-NC group and Hypoxia + anti-miR-4640-5p group were administrated with anti-NC and anti-miR-4640-5p intratracheally, respectively, while Normoxia group and Hypoxia group were given equal volume of PBS. Pulmonary hypertension model was established by exposing the rats in Hypoxia group, Hypoxia + anti-NC group and Hypoxia + anti-miR-4640-5p group to 10% O_2_ for 8 h per day for 4 weeks. After the rats were anesthetized with 30 mg/kg sodium barbital, the abdominal incision was made through the xiphoid process, and the scalp needle was inserted into the right ventricle from the abdominal cavity through the diaphragm to measure the right ventricular systolic pressure (RVSP) and Cardiac output (CO) using a pressure transducer catheter (Millar Instruments).

### Reverse-transcribe quantitative polymer chain reaction (RT-qPCR)

Total RNA was extracted from lung tissue samples and PASMCs using TRIzol reagent (AccuRef Scientific, China) and then reverse transcribed to cDNA using Accuref 1^st^ Strand cDNA synthesis kits (#RM0011, AccuRef Scientific, China) following the manufacturer's recommendations. The total RNA concentration was measured using a NanoDrop spectrophotometer. Quantitative real-time PCR was carried out on Applied Biosystems 7300 real-time PCR system (Applied Biosystems, USA) using the Accuref qPCR SYBR Green Mixture (#RM0031M, AccuRef Scientific). U6 RNA was used as internal control, and the relative expression values were normalized using Ct method (2^−△△Ct^). The primer sequences were listed in Table 1.

**Table 1:** Primers used for qPCR

Gene	Primers (5′ → 3′)	
miR-4640-5p	Forward	TGGGCCAGGGAGCAGCTGGUGGG
	Reverse	Universal oligo dT primer
U6	Forward	CTCGCTTCGGCAGCACA
	Reverse	Universal oligo dT primer
DUSP13	Forward	TTTCATAGGAGATGCGGCCA
	Reverse	CACTGCTGCCGTAGAAGTCA
TMEM184B	Forward	ATCACATGCCACCAGCCCA
	Reverse	GCTGCTCTACCACAGTCCTC
CD209	Forward	TGCTGAGGAGCAGAACTTCC
	Reverse	TACTGCTTGAAGCTGGGCAA
ORAI2	Forward	CGTATAAATGACCTGCCTGGCT
	Reverse	AGGAGCAGAGGGGTCGATAG
AGO1	Forward	CACGCTGGACTTCACAGTCT
	Reverse	CCCGCAGCTGCTCCC
VAMP3	Forward	AGTTAGACGACCGTGCAGAC
	Reverse	CCGATTGCCCACATCTTGC
MYO1E	Forward	ACTGGGAGGAAAGCAGGGTA
	Reverse	AGATGCGCCGATAGGCATAG
NOS1	Forward	ATTTATGCCGCGTTTCCAGC
	Reverse	AGGCATCATGAGCCCGTC
TFCP2L1	Forward	TTCCAGCCATGCTCTTCTGG
	Reverse	CGAGCACATCACGCAGGTA
GAPDH	Forward	ATGGGGAAGGTGAAGGTCG
	Reverse	GGGGTCATTGATGGCAACAATA

### Western blot

Cultured PASMCs were lysed in RIPA buffer (HAT, WB053) with proteinase inhibitor (Roche, Switzerland). Equal amounts of protein were separated by SDS-PAGE and transferred onto PVDF membrane (Immobilon-P, IPRH00010, China). The membranes were blocked with 5% non-fat milk in PBST, followed by incubation with primary antibodies (β-actin, Proteintech 20536-1-AP; mTOR, MERCK, T2949-200UL; p-mTOR (pSer 2448), MERCK, SAB4504476-100UG; p-S6, Abcam, Y179; GAPHD, Proteintech, 60004-1-Ig; anti-NOS1, Santa Cruz, SC-5302) overnight at 4 °C. Subsequently, the membranes were further incubated with a secondary antibody conjugated with HRP (anti-Rabbit IgG, Biosharp, BL003A). The protein levels were visualized by using a Western blot ECL kit (#AP0082, AccuRef Scientific, China).

### Luciferase reporter assay

PASMCs were seeded into 24-well plates. Luciferase reporter vector psiCHECK2 (Promega, USA) containing WT or mutant 3′-UTR of NOS1 was transiently co-transfected with miR-4640-5p mimics or NC into PASMCs cells. The relative luciferase activity was analyzed by Dual-luciferase reporter assay system (Promega, GloMax20/20, USA).

### Statistical analysis

All results were shown as mean ± SD. The statistical analysis was performed using two-tailed student t test or ANOVA with GraphPad Prism V8 software (Prism, USA). A p value < 0.05 was considered as statistical significant.

## Results

### MiR-4640-5p is highly expressed in the lung tissues of COPD-PH patients

**Fig. 1:**
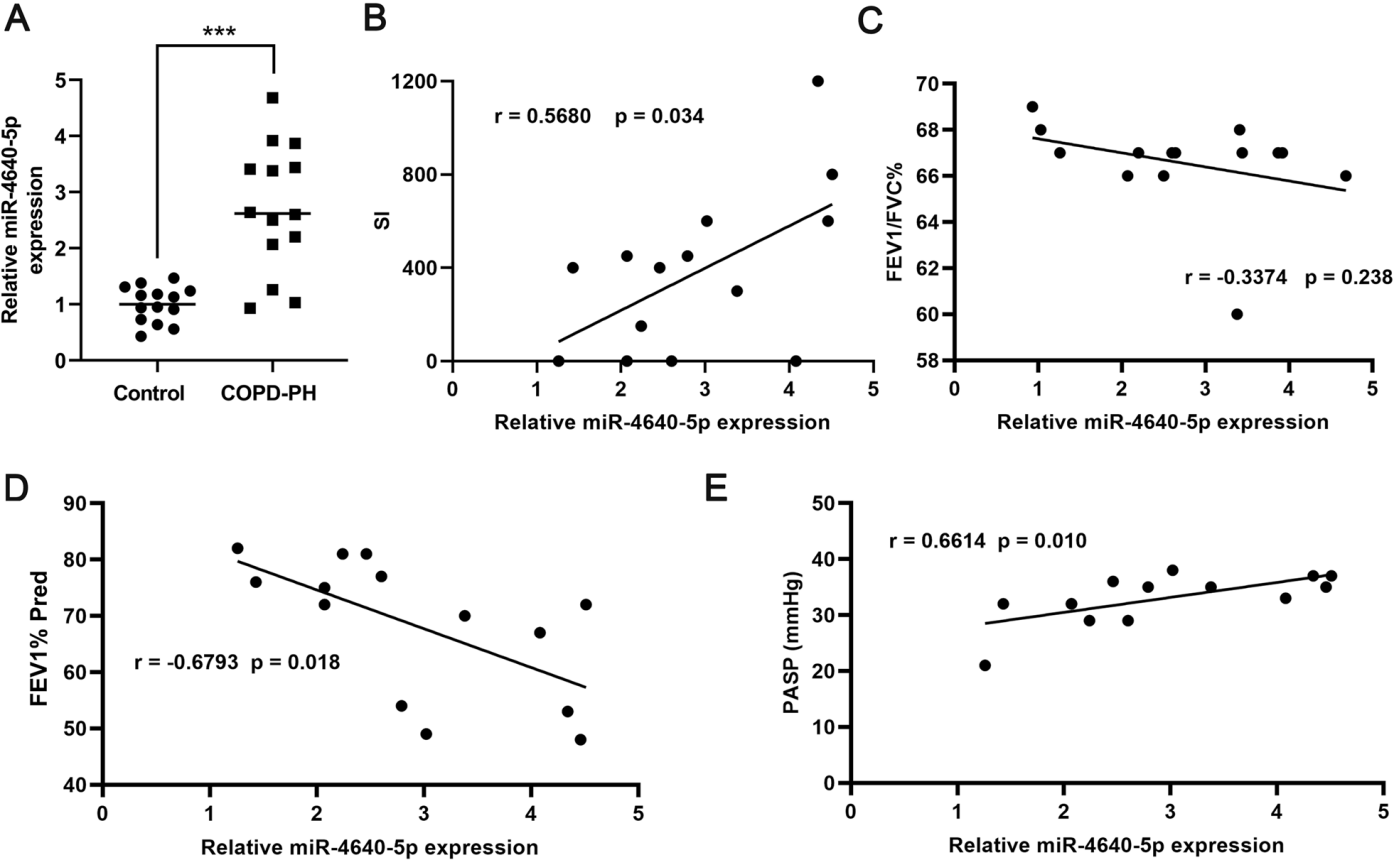
MiR-4640-5p is highly expressed in the lung tissues of COPD-PH patients and correlated with severity of COPD-PH. **A** The expression of miR-4640-5p was examined in the lung tissues of COPD-PH patients (n = 14) and healthy controls (n = 14) by RT-qPCR. **B**–**E** Pearson analysis was performed to analysis the correlation between miR-4640-5p expressions and the smoking index (SI), FEV1/FVC%, FEV1% Pred, and pulmonary artery systolic pressure (PASP). *** p < 0.001

The expression of miR-4640-5p was examined in the lung tissues of COPD-PH patients and normal volunteers by RT-qPCR. As shown in Fig. 1A, miR-4640-5p expression was significantly higher in the lung tissue of COPD-PH patients. Pearson correlation analysis found that the expression of miR-4640-5p was positively associated with the smoking index (SI, r = − 0.5480, p = 0.042, the smoking index is a unit for measuring cigarettes consumption over a long period and was calculated using the following formula: smoking index = the number of cigarettes smoked per day (CPD) × years of tobacco use.) (Fig. 1B). Moreover, the expression levels of miR-4640-5p were negatively correlated with FEV1% Pred, but not FEV1/FVC ratio (Fig. 1C, D). However, miR-4640-5p expression was significantly correlated with the pulmonary artery systolic pressure (PASP) (Fig. 1E).

### Inhibition of miR-4640-5p suppresses cell proliferation and migration of PASMC via regulating mTOR/S6 signaling

**Fig. 2:**
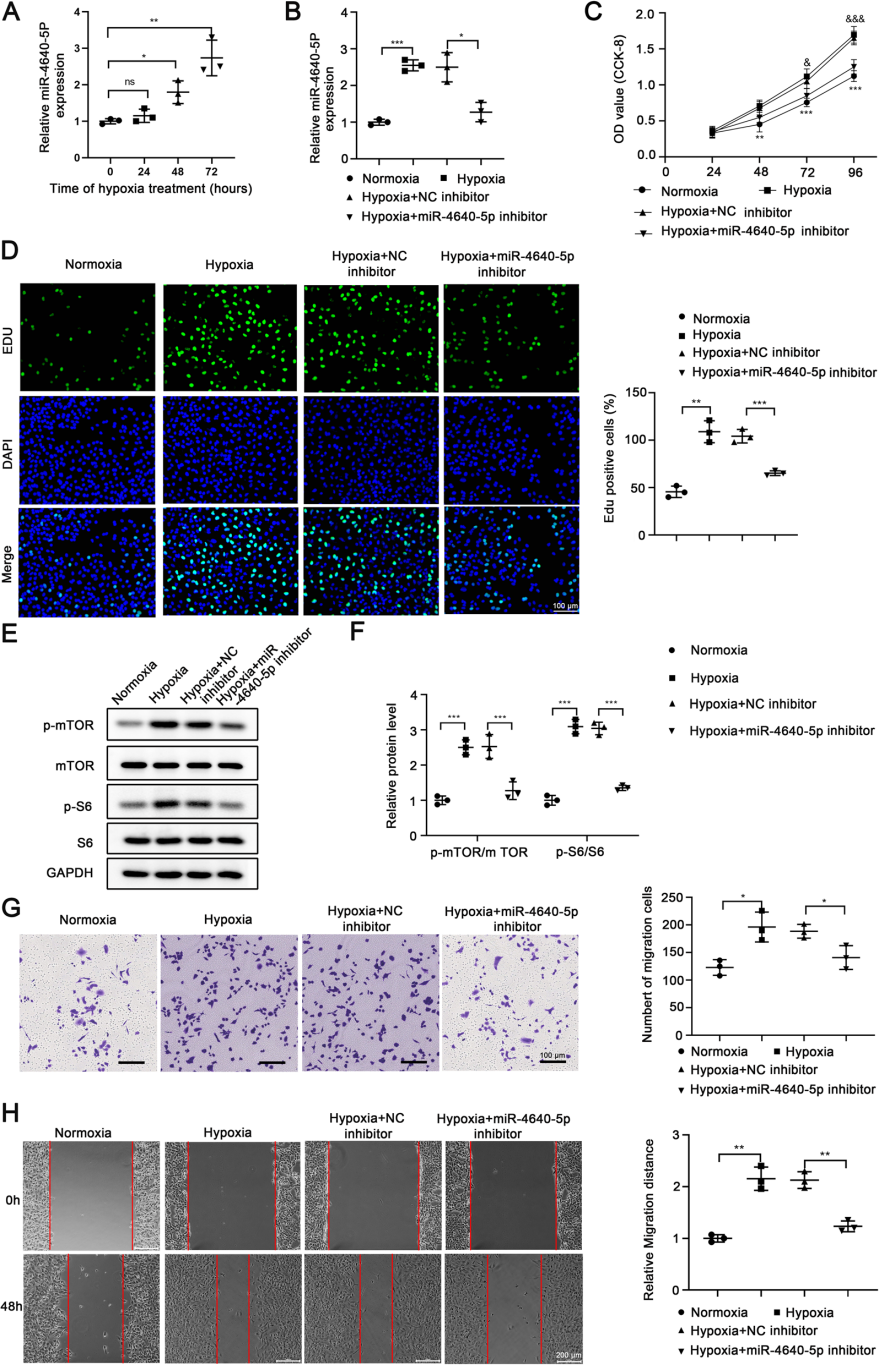
Inhibition of miR-4640-5p suppresses cell proliferation and migration of PASMC via regulating mTOR/S6 signaling. **A** PASMCs were cultured under hypoxia condition for indicated time and the miR-4640-5p expression was analyzed by RT-qPCR. **B**–**G** PASMCs were cultured under normoxia or hypoxia condition, transfected with miR-4640-5p inhibitor or negative control (NC). **B** The relative expression of miR-4640-5p in PASMCs was analyzed by RT-qPCR. **C** Cell proliferation was analyzed by CCK-8 assay. **D** DNA synthesis was analyzed by EdU incorporation assay. **E**, **F** Protein expression levels of p-mTOR, mTOR, p-S6, and S6 in PASMCs were analyzed by western blot. β-actin was used as an internal control. PASMC migration was evaluated by transwell assay (**G**) and wound-healing assay (**H**). All experiments were independently repeated for at least three times. *p < 0.05, **p < 0.01, ***p < 0.001

To further investigate the expression and function of miR-4640-5p in COPD-PH, PASMCs were cultured under hypoxia condition for different time and the expression of miR-4640-5p was analyzed. The results showed that the expression of miR-4640-5p was enhanced in PASMCs cultured under hypoxia, with the highest levels at 72 h post hypoxia treatment (Fig. 2A). Compared with normoxia condition, hypoxia treatment significantly enhanced miR-4640-5p expression while miR-4640-5p inhibitor markedly suppressed miR-4640-5p levels in PASMCs (Fig. 2B). Functionally, we demonstrated that hypoxia treatment remarkably enhanced cell proliferation and DNA incorporation and inhibition of miR-4640-5p significantly suppressed cell growth in PASMCs (Fig. 2C, D). Mammalian target of rapamycin (mTOR) signaling is a central hub regulating cell proliferation, autophagy and apoptosis [18]. Hypoxia treatment of PASMCs enhanced mTOR/S6 signaling (Fig. 2E, F). In the contrast, miR-4640-5p inhibitor could suppress the phosphorylation of mTOR and S6 in PASMCs (Fig. 2E, F). Furthermore, transwell assay and wound-healing assay revealed that hypoxia treatment enhanced PASMC cell migration while inhibition of miR-4640-5p decreased the capability of cell migration in PASMCs (Fig. 2G, H).

### NOS1 is a downstream direct target of miR-4640-5p

**Fig. 3:**
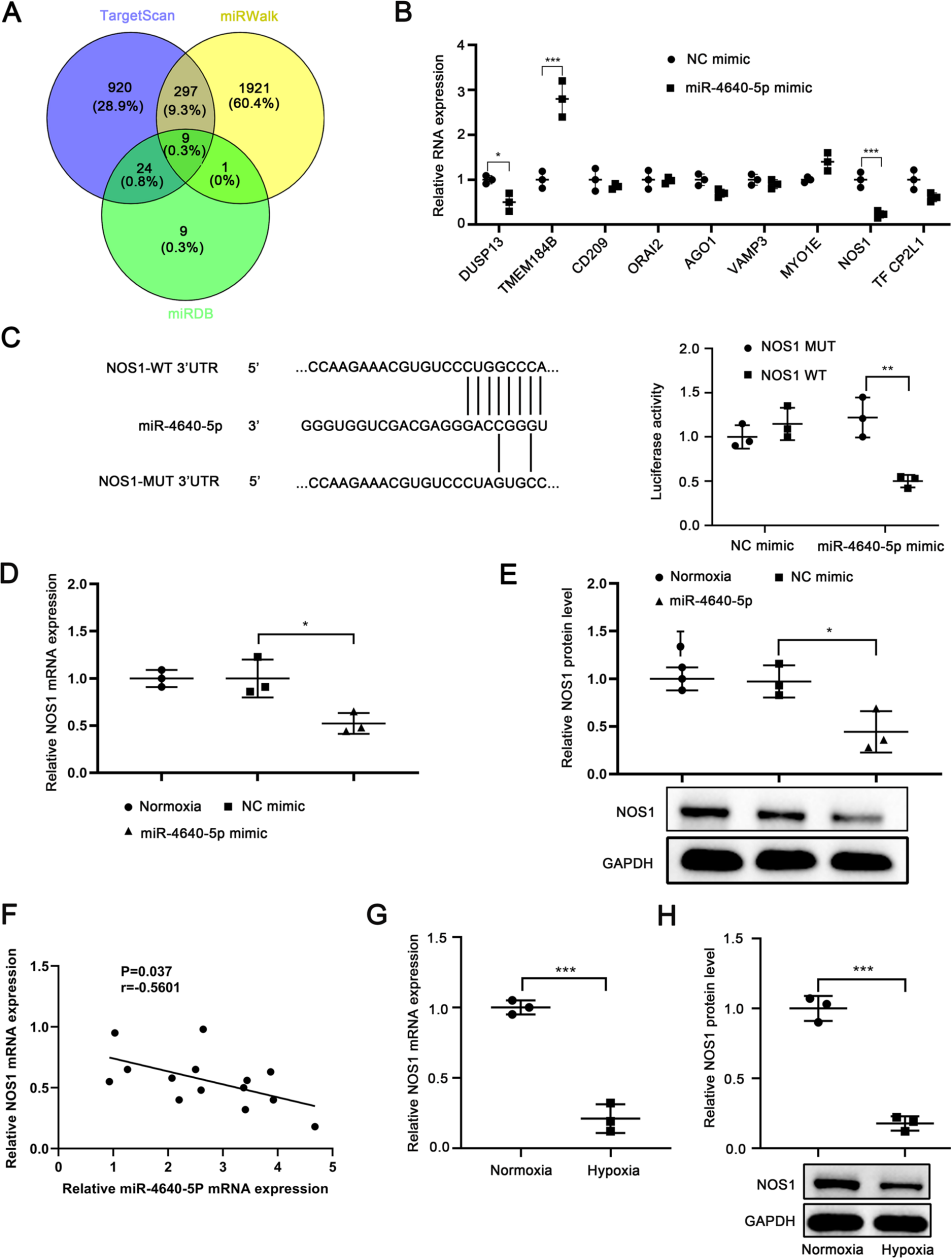
NOS1 is a downstream direct target of miR-4640-5p. **A** A Venn diagram showed the numbers of potential target genes of miR-4640-5p predicted by TargetScan, miRDB, and miRWalk. **B** PASMCs were transfected with miR-4640-5p mimic or NC. The relative mRNA expression levels of commonly predicted genes were analyzed by RT-qPCR 48 h later. **C** Diagrams showed the putative binding sites between miR-4640-5p and corresponding wild type (WT) or mutant (MUT) sites of NOS1. PASMCs were co-transfected with reporter vector containing WT or Mut 3’-UTR of NOS1, together with NC or miR-4640-5p mimic. The dual luciferase activity was analyzed in PASMCs at 48 h post transfection. **D** The mRNA and (**E**) protein expression levels of NOS1 in PASMCs cultured under normoxia or hypoxia, transfected with miR-4640-5p mimic or NC. **F** Pearson analysis of the correlation between NOS1 mRNA expression and miR-4640-5p expression in lung tissue from COPD-PH patients (n = 14). **G**, **H** The mRNA (**G**) and protein (**H**) expression levels of NOS1 in PASMCs cultured under normoxia or hypoxia condition were analyzed by RT-qPCR and western blot. All experiments were independently repeated for at least three times. *p < 0.05, **p < 0.01, ***p < 0.001

By using three different bioinformatics analysis tools (TargetScan, mRDB, and miRWalk), we identified 9 genes as potential targets of miR-4640-5p (Fig. 3A). Among these 9 genes, overexpression of miR-4640-5p in PASMCs significantly suppressed the expression of DUSP13 and NOS1 (Fig. 3B). Further analysis suggested that miR-4640-5p had the complementary binding sequences against 3′-UTR of NOS1 (Fig. 3C). Luciferase reporter assay demonstrated that miR-4640-5p bound to the 3′-UTR of NOS1 and significantly inhibited the luciferase activity in PASMCs transfected with luciferase reporter vector containing WT 3’-UTR of NOS1, but not mutated 3’-UTR of NOS1 (Fig. 3C). Under normoxia condition, we found that overexpression of miR-4640-5p could markedly decreased the mRNA and protein levels of NOS1 (Fig. 3D, E). Intriguingly, the miR-4640-5p expression levels were negatively associated with NOS1 mRNA expression levels in COPD-PH patients (Fig. 3F). Moreover, hypoxia treated PASMCs had significantly lower levels of NOS1 mRNA and protein compared with that in normoxia condition (Fig. 3G, H).

### Overexpression of NOS1 antagonizes the effect of miR-4640-5p in regulating PASMC cell proliferation and migration

**Fig. 4:**
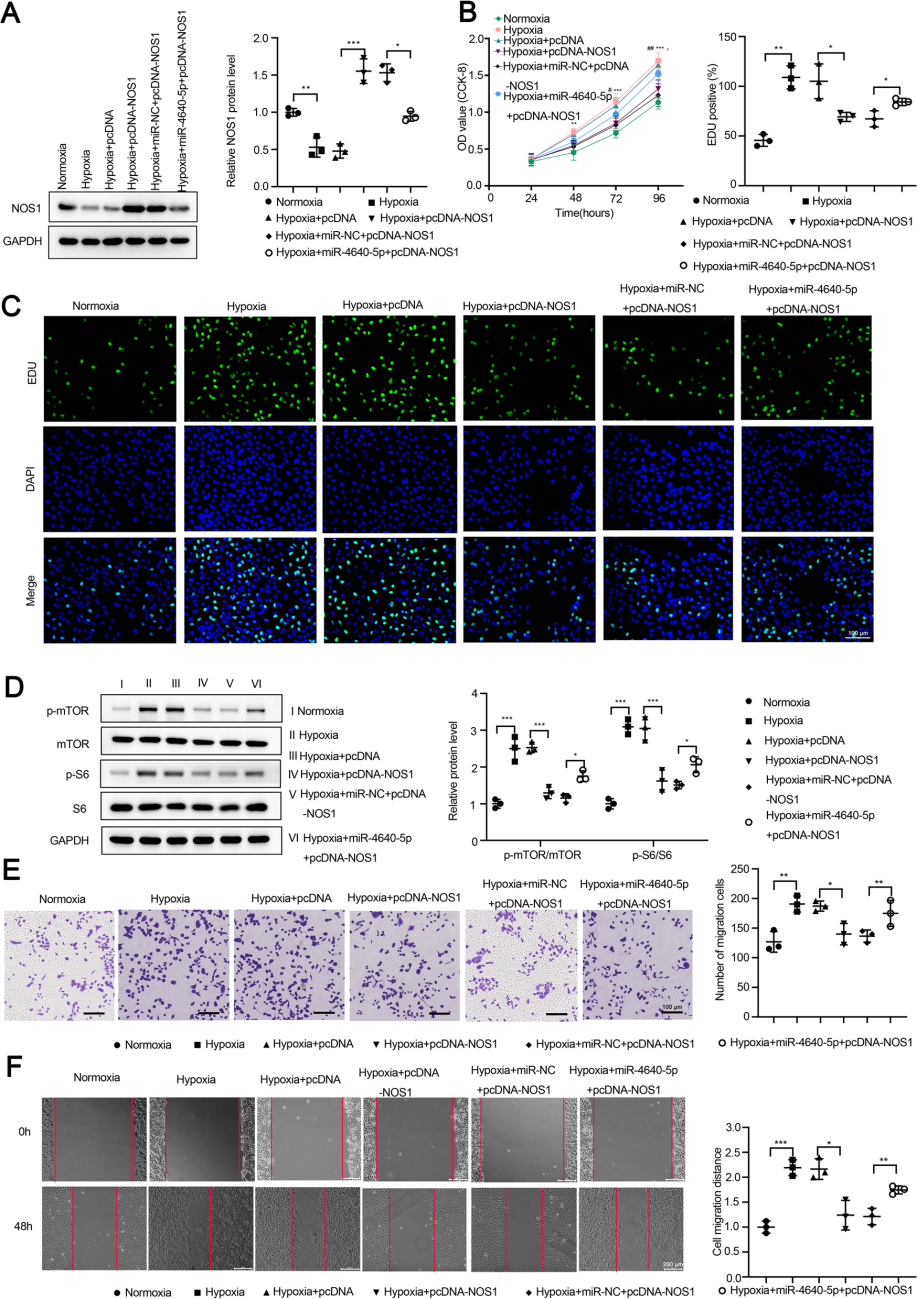
Overexpression of NOS1 antagonizes the effect of miR-4640-5p in regulating PASMC cell proliferation and migration. PASMCs were cultured under normoxia or hypoxia condition, transfected with pcDNA empty vector, pcDNA-NOS1, miR-NC + pcDNA-NOS1, or miR-4640-5p mimic + pcDNA-NOS1. **A** The relative expression of NOS1 in PASMCs was analyzed by western blot. **B** Cell proliferation was analyzed by CCK-8 assay. **C** DNA synthesis was analyzed by EdU incorporation assay. **D** Protein expression levels of p-mTOR, mTOR, p-S6, and S6 in PASMCs were analyzed by western blot. β-actin was used as an internal control. PASMC migration was evaluated by transwell assay (**E**) and wound-healing assay (**F**). All experiments were independently repeated for at least three times. *p < 0.05, **p < 0.01, ***p < 0.001

To further study the functional relationship between miR-4640-5p and NOS1, we overexpressed NOS1, with or without miR-4640-5p mimics in PASMCs (Fig. 4A). Hypoxia treatment decreased the expression of NOS1, while overexpression of NOS1 antagonized the effect of miR-4640-5p and restored NOS1 expression levels in PASMCs (Fig. 4A). Consistently, hypoxia treatment enhanced PASMC cell proliferation and DNA incorporation. Overexpression of NOS1 inhibited cell proliferation, which could be partially reversed by miR-4640-5p mimics (Fig. 4B, C). In addition, NOS1 overexpression inhibited the p-mTOR and p-S6, which could be partially restored by miR-4640-5p overexpression (Fig. 4D). Similarly, hypoxia treatment enhanced cell migration and overexpression of NOS1 dampened the migration capability of PASMCs, which could be partially rescued by miR-4640-5p overexpression (Fig. 4E, F).

### MiR-4640-5p/NOS1 regulates mitochondrial dynamics in PASMCs

**Fig. 5:**
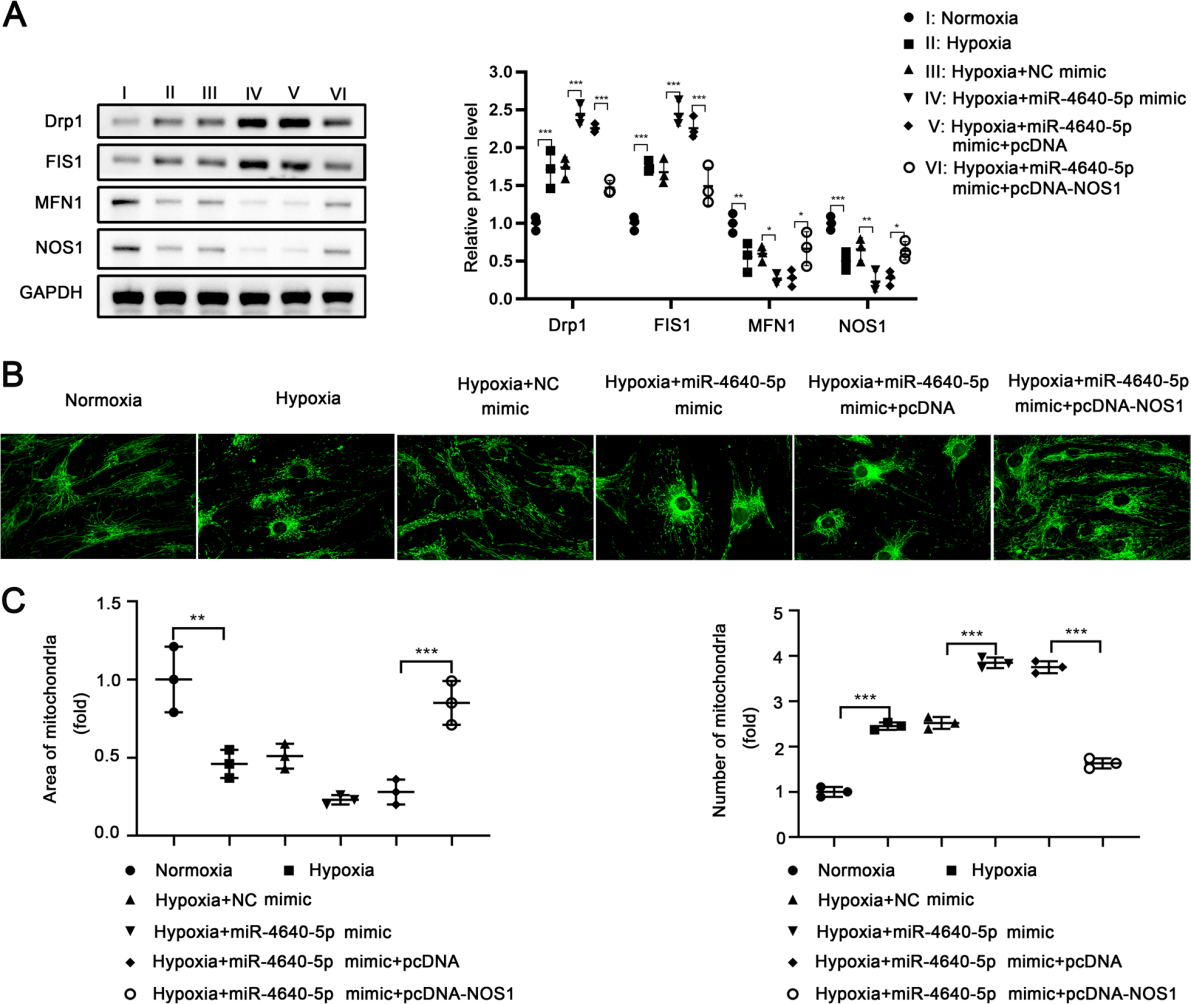
MiR-4640-5p/NOS1 regulates mitochondrial dynamics in PASMCs. PASMCs were cultured under normoxia or hypoxia condition, transfected with pcDNA empty vector, pcDNA-NOS1, miR-NC + pcDNA-NOS1, or miR-4640-5p mimic + pcDNA-NOS1. **A** The protein expression of Drp1, FIS1, MFN1, NOS1 and GAPDH in PASMCs was analyzed by western blot. **B**, **C** Immunofluorescence staining of mitochondria in PASMCs with different treatment was performed. The area of mitochondria and number of mitochondria was analyzed. All experiments were independently repeated for at least three times. *p < 0.05, **p < 0.01, ***p < 0.001

Mitochondrial dysfunction has been reported to participate in COPD-PH pathobiology [19]. We examined the mitochondrial dynamics in PASMCs treated with hypoxia and transfected with miR-4640-5p mimics or NOS1 overexpression vector. As shown in Fig. 5A, hypoxia treatment enhanced the expression of the dynamin 1-like protein (Drp1) and mitochondrial fission 1 (FIS1), but decreased mitofusin 1 (MFN1) and NOS1 expression. In addition, overexpression miR-4640-5p further enhanced Drp1/FIS1 expression and suppressed MFN1/NOS1 expression, while the regulation mediated by miR-4640-5p could be partially antagonized by NOS1 overexpression (Fig. 5A). Consistently, hypoxia treatment or miR-4640-5p overexpression led to reduced area of mitochondria but increased mitochondria numbers, indicating enhanced cell cycle progression and cell proliferation (Fig. 5B, C). However, overexpression of NOS1 restored the area of mitochondria and decreased the number of mitochondria (Fig. 5B, C).

### Inhibition of miR-4640-5p ameliorates hypoxia-induced PAH in rat

**Fig. 6:**
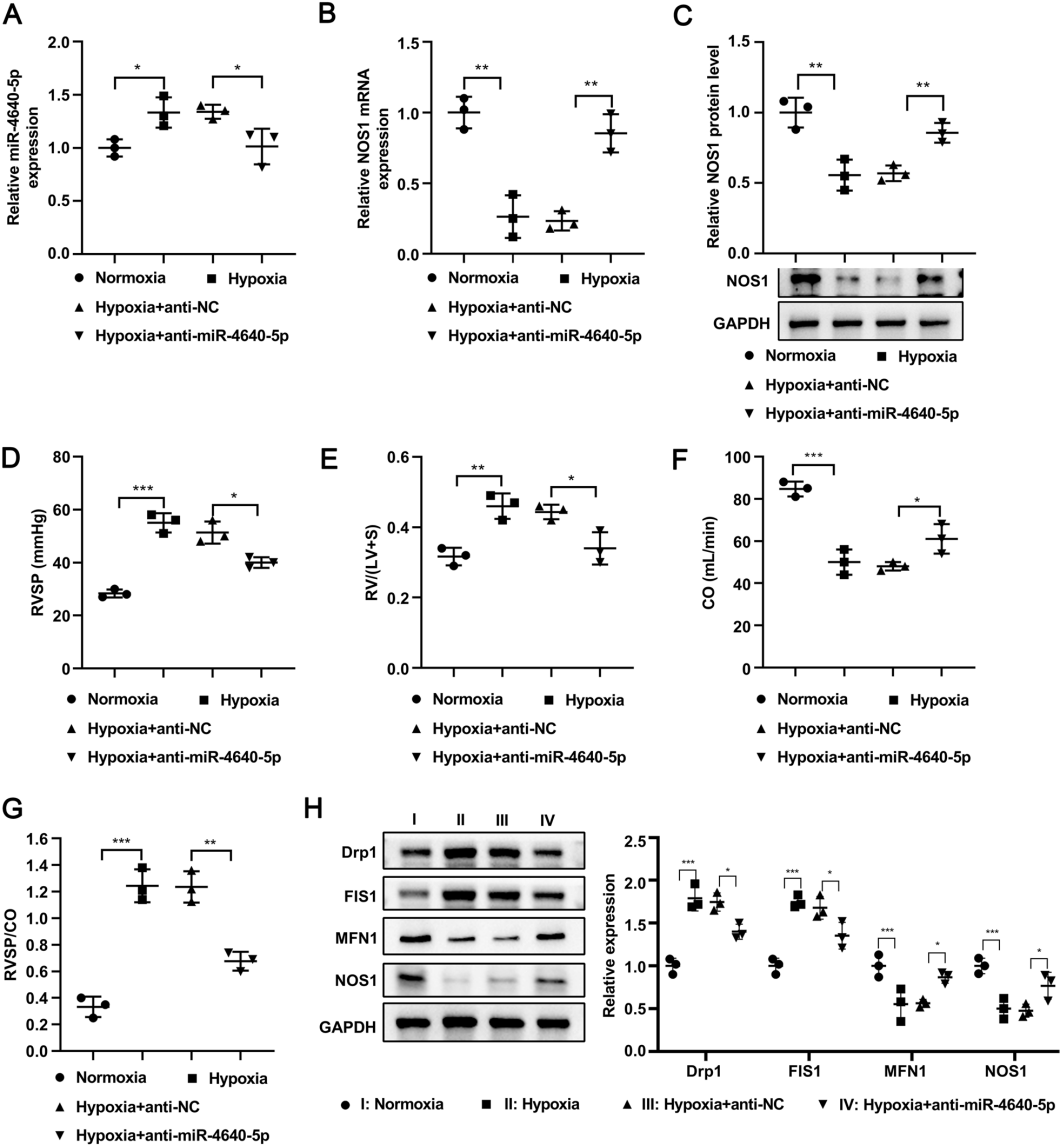
Inhibition of miR-4640-5p ameliorates hypoxia-induced PAH in rat. Sprague–Dawley rats were randomly divided into four groups (n = 5): Normoxia group, Hypoxia group, Hypoxia + anti-NC group, Hypoxia + anti-miR-4640-5p group. Pulmonary hypertension model was established by exposing the rats in Hypoxia group, Hypoxia + anti-NC group and Hypoxia + anti-miR-4640-5p group to 10% O_2_ for 8 h per day for 4 weeks. After terminal harvest, the relative expression of miR-4640-5p (**A**), NOS1 mRNA (**B**), and NOS1 protein (**C**) in rat lung tissues was analyzed. **D**–**G** After the rats were anesthetized with 30 mg/kg sodium barbital, the abdominal incision was made through the xiphoid process, and the scalp needle was inserted into the right ventricle from the abdominal cavity through the diaphragm to measure and calculate the (**D**) RVSP, (**E**) RV/(LV + S), (**F**) CO, and (**G**) RVSP/CO. **H** The protein expression of Drp1, FIS1, MFN1, NOS1 and GAPDH in PASMCs was analyzed by western blot. Western blot experiments were independently repeated for at least three times *p < 0.05, **p < 0.01, ***p < 0.001

To further investigate the function of miR-4640-5p in vivo, we established the hypoxia-induced PAH rat model by exposing Sprague–Dawley (SD) rat in hypoxia chamber (10% O_2_, 8 h per day) for 4 weeks. SD rats were treated with PBS under normoxia or hypoxia, or administrated with anti-miRNA negative control or anti-miR-4640-5p. The results showed that hypoxia exposure enhanced miR-4640-5p expression while down-regulated the mRNA and protein levels of NOS1 in rat PASMCs (Fig. 6A–C). Inhibition of miR-4640-5p enhanced NOS1 mRNA and protein expression in rat PASMCs (Fig. 6A–C). Functionally, hypoxia treatment resulted in enhanced right ventricular systolic pressure (RVSP) and Fulton index (RV/(LV + S)), with decreased cardiac output (CO) (Fig. 6D–F). Treatment with anti-miR-4640-5p ameliorated the severity of PH, showing decreased RVSP and Fulton index and increased CO (Fig. 6D–F). In addition, the RVSP/CO ratio was significantly enhanced under hypoxia treatment but markedly decreased with anti-miR-4640-5p treatment (Fig. 6G). Consistent with the in vitro results, hypoxia treatment decreased FIS1 and NOS1 expression in rat PASMCs while enhanced Drp1 and FIS1 expression (Fig. 6H). Inhibition of miR-4640-5p partially reversed the effect of hypoxia treatment (Fig. 6H).

## Discussion

The pathophysiology of COPD-PH is multifactorial and heterogeneous [20]. Mounting evidence show that miRNAs play critical functions in COPD-PH [21–23]. Here, we identified miR-4640-5p was highly expressed in the lung tissues of COPD-PH patients, which was correlated with more severe COPD-PH. Inhibition of miR-4640-5p suppressed cell proliferation and migration of PASMC in vitro and ameliorated PH with reduced right ventricular systolic pressure and Fulton index in hypoxia-induced rat PH in vivo. The miR-4640-5p/NOS1 axis could be utilized as a potential diagnostic biomarker and therapeutic target for COPD-PH patients.

The function of miR-4640-5p has been investigated in multiple cancer types such as laryngeal squamous cell carcinoma, endometrial cancer and palillary thyroid cancer [24–26]. In NSCLC, lncRNA OGFRP1 functions as an oncogene via sponging miR-4640-5p [13]. However, the expression profile and function of miR-4640-5p in lung tissues of COPD-PH patients have not been addressed. Our results showed higher miR-4640-5p expression in COPD-PH lung tissues, which was correlated with more severe PH (Fig. 1). PASMC cell hyper-proliferation is an essential cause of vessel intimal thickening and pulmonary vascular remodeling while PASMC proliferation could be regulated by chronic hypoxia exposure [27]. Consistently, we found that hypoxia treatment enhanced miR-4640-5p expression and promoted PASMC cell proliferation and migration, which was associated with enhanced mTOR/S6 signaling.

MicroRNA-mRNA network contributes to the pathogenesis of idiopathic pulmonary arterial hypertension [28]. In this study, bioinformatics analysis indicated that NOS1 might be a direct downstream target of miR-4640-5p. It has been reported that the mRNA and protein expression of NOS1 was significantly higher in lung tissues of smokers with COPD compared with nonsmoker controls [17]. We validated the interaction between miR-4640-5p and NOS1 by using luciferase reporter assay, while overexpression of NOS1 could functionally reverse the effect of miR-4640-5p overexpression. However, due to the multiple-to-multiple regulation between miRNAs and target genes, there might be other downstream target genes of miR-4640-5p involved in the regulation of pathogenesis of COPD-PH, which requires further investigation.

Intriguingly, mitochondrial dysfunction has been reported to participate in COPD-PH pathobiology [19]. Similarly, we found that hypoxia treatment or miR-4640-5p overexpression led to reduced area of mitochondria but increased mitochondria numbers, indicating enhanced cell cycle progression and cell proliferation, while overexpression of NOS1 restored the area of mitochondria and decreased the number of mitochondria. The function of miR-4640-5p was also evaluated in a hypoxia-induced PH rat model in vivo, suggesting that inhibition of miR-4640-5p could ameliorate the hypoxia-induced PH development in rat.

## Conclusions

In summary, our findings suggest that inhibition of miR-4640-5p could suppress PASMC cell proliferation and migration, ameliorate hypoxia-induced PH via targeting NOS1 and regulating mTOR/S6 signaling. Our results might provide a potential novel approach for COPD-PH treatment.

## Supplementary Information

**Additional file 1. Table S1.** Information about the patient specimens of experimental group.

**Additional file 2. Table S2.** Information about the patient specimens of control group.

**Additional file 3. Table S3.** Statistical information about the patient specimens of control and experimental group.

## Data Availability

The data of current study are available from the corresponding author upon reasonable request.
